# Effect of the Inoculum-to-Substrate Ratio on Putative Pathogens and Microbial Kinetics during the Batch Anaerobic Digestion of Simulated Food Waste

**DOI:** 10.3390/microorganisms12030603

**Published:** 2024-03-18

**Authors:** Saanu Victoria Otite, Bhushan P. Gandhi, Esther Agyabeng Fofie, Alfonso José Lag-Brotons, Lawrence I. Ezemonye, Alastair D. Martin, Roger W. Pickup, Kirk T. Semple

**Affiliations:** 1 Lancaster Environment Centre, Lancaster University, Library Avenue, Lancaster LA1 4YQ, UK; s.obatusin@lancaster.ac.uk (S.V.O.); e.agyabengfofie@lancaster.ac.uk (E.A.F.); 2 Engineering Department, Lancaster University, Gillow Avenue, Lancaster LA1 4YW, UK; b.gandhi@lancaster.ac.uk (B.P.G.); a.martin1@lancaster.ac.uk (A.D.M.); 3 Centre for Global Eco-Innovation Nigeria, University of Benin, Benin City P.O. Box 300313, Edo State, Nigeria; ezemslaw@yahoo.com; 4 Vice Chancellor’s Office, Igbinedion University Okada, Benin City P.O. Box 0006, Edo State, Nigeria; 5 Division of Biomedical and Life Sciences, Faculty of Health and Medicine, Lancaster University, Furness Building, Lancaster LA1 4YG, UK; r.pickup@lancaster.ac.uk

**Keywords:** anaerobic digestion, fluorescent-labelled *Escherichia coli*, quantitative PCR, pathogen reduction, *Enterococcus* resistance, Sanger sequencing

## Abstract

The effects of the inoculum (anaerobic digestion effluent) to substrate (simulated food waste) ratio (ISR) 4.00 to 0.25 on putative pathogens and microbial kinetics during batch mesophilic anaerobic digestion were investigated. Red fluorescent protein labelled (RFPAKN132) *Escherichia coli* JM105 was introduced as a marker species, and together with the indigenous *Clostridium* sp., *Enterococcus* sp., *Escherichia coli*, and total coliforms were used to monitor pathogen death kinetics. Quantitative polymerase chain reaction was also used to estimate the bacterial, fungal, and methanogenic gene copies. All the ISRs eliminated *E. coli* and other coliforms (4 log_10_ CFU/mL), but ISR 0.25 achieved this within the shortest time (≤2 days), while ISR 1.00 initially supported pathogen proliferation. Up to 1.5 log_10_ CFU/mL of *Clostridium* was reduced by acidogenic conditions (ISR 0.25 and 0.50), while *Enterococcus* species were resistant to the digestion conditions. Fungal DNA was reduced (≥5 log_10_ copies/mL) and was undetectable in ISRs 4.00, 2.00, and 0.50 at the end of the incubation period. This study has demonstrated that ISR influenced the pH of the digesters during batch mesophilic anaerobic digestion, and that acidic and alkaline conditions achieved by the lower (0.50 and 0.25) and higher (4.00 and 2.00) ISRs, respectively, were critical to the sanitisation of waste.

## 1. Introduction

Anaerobic digestion (AD) is a biological process that is used in the treatment and stabilisation of biodegradable wastes in wastewater treatment plants for municipal solid waste, as well as food, agricultural, and industrial wastes [1,2]. The AD process generates biogas and a residue known as digestate, which can be used as a soil conditioner or biofertiliser, depending on the amount of nutrient it contains [3]. Food wastes are a source of nutrient-rich and biodegradable material, having a high solid content, and they are suitable for co-digestion with other substrates. This makes food waste an ideal feedstock for AD [4,5,6].

Although it has been reported that AD reduces or eliminates human pathogens and antibiotic-resistant genes present in food wastes and other organic wastes [2,7,8,9], safety concerns remain with the recycling of digestate through its application to soil. This is because certain pathogenic species may persist in the digestate despite AD treatment [10,11]. This has raised questions on the efficacy of the AD process in sanitizing food wastes which could contain high concentrations of potentially pathogenic enteric species such as *Salmonella enterica*, *Escherichia coli*, *Enterococcus faecalis*, and *Shigella* spp. [12,13,14]. These organisms may exhibit different survival capabilities while in the natural environment and could pose public health risks. They can contaminate the soil and crops, as well as leach into surface and underground water, resulting in food and water-borne diseases [15]. Therefore, strict regulations are required concerning the use of sanitised digestate in agriculture.

Factors that influence the performance and efficacy of the AD process in eliminating pathogens include temperature, pH, retention time, digester configuration, microbial species, and presence of intermediates such as volatile fatty acids (VFAs) and ammonia [16]. The temperature of digestion is an important factor in pathogen inactivation during AD. The temperature is characterised as being in the psychrophilic, mesophilic, or thermophilic range. Thermophilic anaerobic digestion was reported to eliminate *Salmonella* Senftenberg and *Enterococcus* spp. During the AD of food waste [8]. Despite the sanitising efficacy of thermophilic AD, mesophilic anaerobic digestion (25–40 °C) is the most common type used due to the modest energy input required and the ease of handling [17]. This mesophilic temperature range also coincides with the optimum temperature of most human pathogens, which tends to support their growth and proliferation. Therefore, mesophilic AD requires other “potent” conditions beyond temperature to achieve the sanitisation of waste.

pH is influenced by the concentration of VFAs and the buffering capacity of the digester. The concentration of VFAs produced can be a function of the initial total solid concentration, which is strongly influenced by the ratio of inoculum to substrate [18]. The complex interplay of these initial conditions in the digester affects the pH, methanogenesis, and rate of pathogen decline during the treatment of wastes. The conditions that favour the growth of methanogenic organisms and thus biogas production also tend to favour the survival of pathogenic species. Most microorganisms grow and survive optimally at a pH of about 7 [19]. Thus, higher deviations from this neutral pH towards acidity or alkalinity can affect pathogen survival during AD and thus aid in the sanitisation of waste. Hence, the control of other parameters such as the inoculum-to-substrate ratio (ISR), pH, and digestion method during the AD process is pertinent.

The ISR determines the rate at which the digester material becomes hydrolysed, as well as the stability of the AD process [20]. Furthermore, Fontana et al. [21] noted that microbial diversity in digesters depends on the inoculum. The inoculum contains acclimatised active microbial species from a previous anaerobic process or a natural anaerobic environment. These species act as starters which help to initiate the digestion process and reduce the lag phase. They should be present in high microbial numbers in the inoculum to enhance the process rate [1]. However, appropriate ISR should be mixed to form the feedstock for the AD process. This is to ensure sufficient starter species, as well as enough substrate to supply the required nutrients for biogas production, because suboptimal ratios of substrate and inoculum can either lead to AD inhibition or an inefficient process.

The effects of ISR on the AD process stability and biogas production have been studied [1,20,22,23,24,25], but there is little work that has been performed on the effect of ISR on pathogen kinetics. Although Arias et al. [26] reported the effect of high total solids on pathogen inactivation during batch mesophilic AD of agricultural residue, and Fagbohungbe et al. [27] investigated the effect of ISR on batch AD of human faecal material, both authors used different feedstock and pathogen detection methods and reported results for only the initial and final pathogen loadings. This research considers the effect of ISR on pathogen and microbial kinetics during batch mesophilic anaerobic digestion of simulated food waste, using culture-based and *q*PCR analyses respectively. This study will aid in determining the upper and lower limits of ISRs, whereby conditions suitable for waste sanitisation can be achieved during mesophilic AD. This work is part of a bigger project, in which the first part containing AD process optimisation and biogas production has been published in Gandhi et al. [25].

## 2. Materials and Methods

### 2.1. Substrate and Inoculum

The substrate was simulated food waste (SFW). Fresh food materials made up of all classes of food (carbohydrate, protein, vitamins, lipids) were purchased from Sainsbury’s PLC, Lancaster, UK (Table S1). The food samples were homogenised using a blender and mixed to obtain the SFW based on the Waste and Resource Action Programme composition of a typical household’s food waste [28]. The inoculum was prepared from anaerobic digester effluent obtained from Cockerham Green Energy Limited biodigester plant (Lancaster, UK) treating livestock and agricultural waste. The anaerobic digestion effluent was sieved through a 2 mm mesh. The simulated food waste and inoculum were stored at −20 °C and 4 °C, respectively, prior to the experimental setup. Detailed characteristics of feedstock can be found in Table S2.

### 2.2. Background Bacterial Enumeration of Substrate and Inoculum

Background bacterial enumeration of the simulated food waste and inoculum was carried out using Nutrient agar, MacConkey agar, Tryptone bile X-glucuronide agar, Brilliance *E. coli* coliform selective medium, Tryptone sulfite neomycin, and bile aesculin agar (All media from Oxoid, UK) to determine total heterotrophic bacteria, Gram-negative enteric bacteria, *Escherichia coli*, coliforms, *Clostridium* sp., and *Enterococcus* sp. respectively. Bacterial isolation comprised serial dilution of samples and inoculation on the aforementioned selective agar plates using the spread plate method. The limit of detection for bacterial enumeration was estimated at 10 CFU/mL, based on the inoculation of undiluted effluent. Plates for the isolation of *Clostridium* were placed in anaerobic jar (ThermoFisher Scientific AnaeroGen^TM^ 2.5 l, Waltham, MA, USA) containing gas generating kit (ThermoFisher Scientific AN0025A). Plates containing media without inoculum were used as the experimental control. All plates were incubated at 37 °C for 18–24 h.

### 2.3. Recovery and Confirmation of Marker Fluorescent-Labelled Escherichia coli Strain

Red fluorescent protein (RFP)-labelled (RFPAKN132) *Escherichia coli* strain JM105 was obtained from Biomedical and Life Sciences laboratory, Lancaster University, and stored as Petri dish culture at 4 °C. The strain carries a mini-Tn7 (Gm) PA1/04/03 DsRedExpress-a delivery plasmid [29]. This plasmid is a pUC derivative expressing resistance to ampicillin, chloramphenicol, and gentamicin antibiotics, which are known effective antibiotics against wild-type *E. coli*. The RFP-labelled strain was recovered from refrigerated solid agar plate using Luria-Bertani broth containing 100 μg/mL each of ampicillin and gentamicin, followed by plating on LB agar with the same antibiotics.

Culture plates were incubated at 37 °C for 24 h. After incubation, cultures were checked for red fluorescence by viewing them in a dark chamber. Pure colonies of fluorescent strain were picked, inoculated into LB broth, and prepared for seeding as a marker species in the anaerobic bioreactors. The concentration of RFP-labelled *E. coli* strain present in culture solution used as inoculum for AD was standardised to 10^8^ CFU/mL by measuring the optical density at OD 600 nm and plate count.

### 2.4. Anaerobic Digestion Setup

The experiment was set up as described by Gandhi et al. [25]. Briefly, the batch bioreactors consisted of five treatments each in duplicate, and controls also in duplicate, totaling twelve reactors. The five treatments were ISR 0.25, 0.50, 1.00, 2.00, and 4.00 on a dry volatile matter basis, and the control had only inoculum without substrate (ISR = ∞). The ratios of 0.25–4.00 were chosen in order to have a lower and upper limit of ratios whereby acidogenesis, which is important for waste sanitization, could be achieved. The contents of all the 5 L capacity bioreactors were made up of deionised water to give an equal working volume of 3.5 L. A 1 mL measure of the marker RFP-labelled *E. coli* JM 105 strain (2.2 × 10^8^ CFU/mL) was mixed with the feedstock and introduced into the AD bioreactor to give a final reactor concentration of 6.2 × 10^4^ CFU/mL. The reactors were sparged with nitrogen gas to generate an anaerobic condition and subsequently incubated at a mesophilic temperature of 37 ± 1.5 °C for 27 d under constant stirring with an in-built U-shaped agitator.

### 2.5. Monitoring the Fate of Marker Escherichia coli and Other Resident Pathogens during AD

Approximately 120 mL of the sample was taken from each bioreactor each day for the first four days, then on alternate days until the tenth day, and also on days 13, 16, 19, and 27. The samples were stored in a freezer at −30 °C prior to analysis for pathogen and microbial dynamics. A sample from each reactor was plated in replicates, giving a total of four determinations per treatment. Culturable aerobic bacteria enumeration was performed using the plate count method on the different selective media as listed in Section 2.2.

### 2.6. pH Determination

The pH of samples was determined according to standard methods [30] using a pH electrode (Mettler Toledo, Columbus, OH, USA). The average pH reading of 4 determinations was recorded.

### 2.7. Molecular Analyses

#### 2.7.1. DNA Extraction and Sanger Sequencing

Total DNA for *q*PCR was extracted from 500 µL of digestate samples collected at 0, 1, and 27 d using Nucleospin soil DNA isolation kit (Macherey-Nagel, Düren, Germany). DNA for Sanger sequencing was extracted from pure culture of uniquely re-occurring bacterial isolates on nutrient agar plates using Quick-DNA bacterial kit (Zymo Research, Tustin, CA, USA). These isolates were present in all the bioreactors during the digestion period. The extracted DNA was quantified using Qubit fluorometer (ThermoFisher Scientific, Oxford, UK), and quality was checked using the Nanodrop 1000 spectrophotometer (ThermoFisher Scientific, UK). The DNA extracted from pure culture isolates was sent to Source Biosciences (Nottingham, UK) for Sanger sequencing. The sequencing was performed using primers targeting the bacteria 16S rRNA gene. The primers were 27F: AGAGTTTGATCMTGGCTCAG, and 1391R: GACGGGCRGTGWGTRCA. The sequences obtained were analysed with the GenBank database (Nucleotide BLAST). Species were assigned based on 100% coverage and identity greater than 98%.

#### 2.7.2. RT-*q*PCR Analysis for Microbial Quantification

The 16S rRNA gene copies of methanogens and bacteria and ITS1 gene copies of fungi were quantified using real-time quantitative polymerase chain reaction (RT-*q*PCR). The primers used were synthesized by ThermoFisher Scientific (UK) (Supplementary Table S1). The 20 µL reaction mixture was made up of 10 µL Platinum SYBR Green *q*PCR SuperMix-UDG (ThermoFisher Scientific, UK), 0.4 µL of Rox dye (final concentration of 50 nM), 0.4 µL of MgCl_2_ (final concentration of 4.0 mM), 2 µL each of forward and reverse primers, 2 µL of DNA template (10 ng/µL), and 5.2 µL of PCR-grade water. Accucal D (Accugen Systems, North Ryde, Australia) at 50, 40, 30, 20, 10, and 0 ng was used as the standard curve calibrator. The reaction was run in ABI 7500 thermocycler (Applied Biosystems, Waltham, MA, USA) with the following run method: 50 °C for 2 min, 95 °C for 2 min, 40 cycles of 95 °C for 15 s; annealing at 60 °C for 30 s; and melt curve analysis of 95 °C for 15 s, 60 °C for 1 min, 95 °C for 15 s and 60 °C for 15 s. The result of the amplification was exported and analysed using the RealCount Software (Version 11.6, Accugen Systems, Australia) to obtain the starting gene copy number.

### 2.8. Secondary Digestion of AD Effluent

The digestate collected after the 27 d incubation was subjected to secondary digestion via in-flask storage to eliminate residual pathogens. Digestate samples were collected from each of the replicate reactors (ISR 1.00) that had the highest terminal pathogen loading. The samples from both reactors were mixed to form a single composite. Approximately 200 mL of the mixture was dispensed into each of two 250 mL capacity Erlenmeyer flasks. The Erlenmeyer flasks were covered with sterile cotton wool and incubated in a controlled temperature room with an average temperature of 25 °C for a further 69 d.

Samples from days 0 and 69 of the secondary incubation were collected on the first and last days of the secondary incubation, respectively, and analysed as described in Section 2.2.

### 2.9. Statistical Analysis

The experiment was carried out in duplicate, and plating was also carried out in duplicate, giving a total of four determinations per treatment. Statistical analysis was performed with IBM SPSS version 28 software. The mean and standard deviation of bacterial counts were determined. One-way analysis of variance (ANOVA) was used to determine the significance of ISR on pathogen decline across the reactors at *p* < 0.05. Tukey’s Honest Post Hoc test was used to compare the significance of means within the ISRs (*p* < 0.05).

## 3. Results

### 3.1. Background Bacterial Species in Substrate and Inoculum

The background bacterial enumeration of the substrate (SFW) and inoculum revealed the presence of culturable bacterial species. The inoculum had a higher total culturable bacteria count compared to the substrate (Table 1). The substrate contained mainly coliforms and *Enterococcus* spp., while the inoculum contained coliforms, *Clostridium* spp., *Enterococcus* spp., and non-lactose fermenting Gram-negative rods. *Escherichia coli* was not detected in either the substrate or the inoculum.

### 3.2. Fate of Indicator and Bacterial Pathogens during AD Using Culture-Dependent Analysis

#### 3.2.1. Marker *E. coli* Strain, Resident *E. coli* Strains and Coliforms

**Figure 1:**
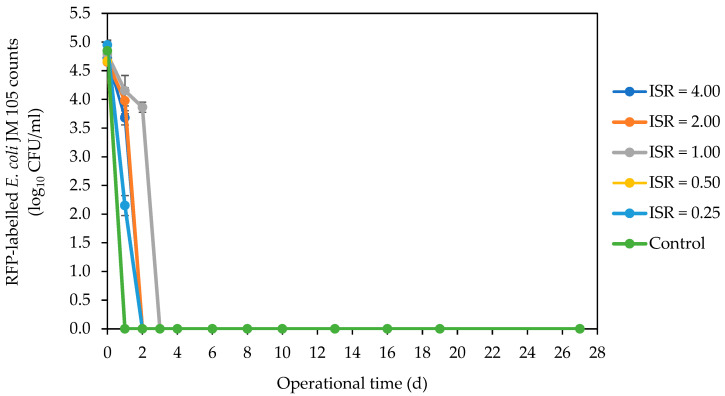
Effect of ISR (4.00, 2.00, 1.00, 0.50, and 0.25) and control on red fluorescent-labelled *Escherichia coli* numbers (log_10_ CFU/mL) during the 27 d anaerobic digestion period. Data points represent the mean and standard deviation of 4 determinations.

**Figure 2:**
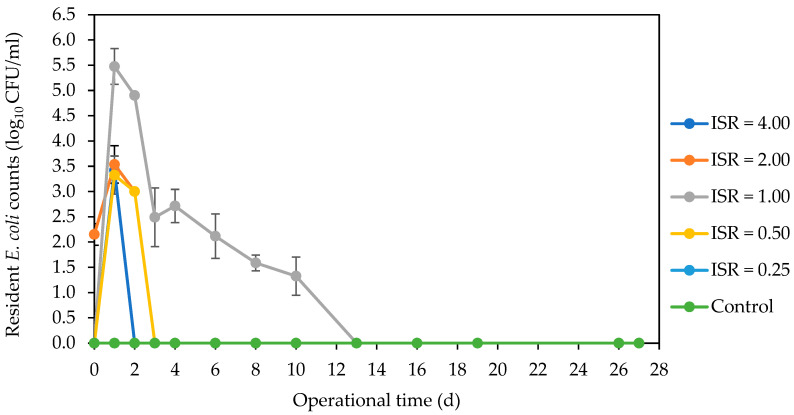
Effect of ISR (4.00, 2.00, 1.00, 0.50, 0.25) and control on resident *Escherichia coli* numbers (log_10_ CFU/mL) during the 27 d anaerobic digestion period. Data points represent the mean and standard deviation of 4 determinations.

**Figure 3:**
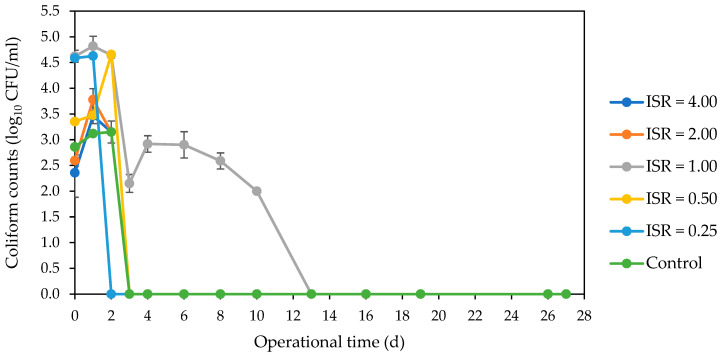
Effect of ISR (4.00, 2.00, 1.00, 0.50, 0.25) and control on coliform numbers (log_10_ CFU/mL) during the 27 d anaerobic digestion period. Data points represent the mean and standard deviation of 4 determinations.

The spiked fluorescent *E. coli* numbers decreased across all ISRs (Figure 1). There was a sharp decline in numbers to below detectable levels in ISR 0.50 and control on day 1. This undetectable *E. coli* level was achieved in ISR 4.00, 2.00, and 0.25 on day 2 and in ISR 1.00 on day 3. The resident *E. coli* strain was not detected in the feedstock (SFW and inoculum) used for this digestion process. But it was detected in ISR 2.00 on day 0, and in the other ISRs except ISR 0.25 on day 1, with over 5 log_10_ CFU/mL in ISR 1.00 (Figure 2). There was complete elimination of this *E. coli* strain in ISR 4.00 as well as in ISR 0.50 and ISR 2.00 on days 2 and 3, respectively. The strain persisted in ISR 1.00 until day 13, when the level went below the limit of detection. Coliforms were eliminated most rapidly in the ISR 0.25, as there were no detectable numbers on the second day of digestion (Figure 3). Complete elimination was also achieved in the control and other ISRs on day 3, except for ISR 1.00, which had coliform counts until d13, when it was below detection.

#### 3.2.2. *Clostridium* spp.

**Figure 4:**
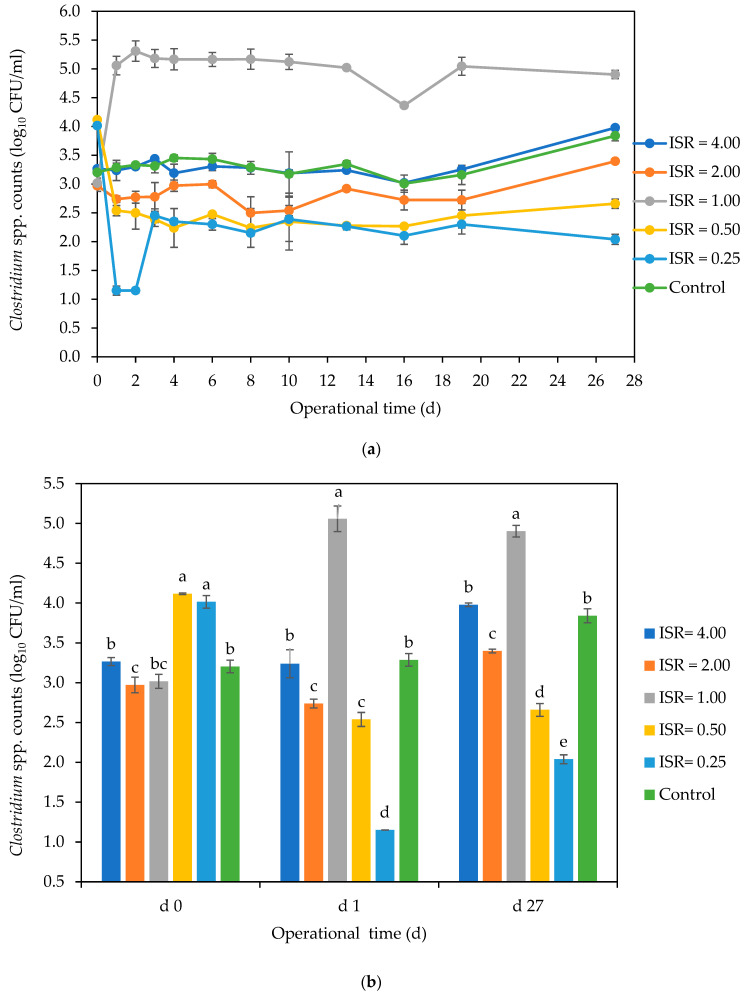
Effect of ISR (4.00, 2.00, 1.00, 0.50, 0.25) and control on *Clostridium* numbers (log_10_ CFU/mL) during the 27 d anaerobic digestion period (**a**) and on days 0, 1 and 27 (**b**). The data points and bars represent the mean and standard deviation of 4 determinations. The letters on the bars are results obtained from Tukey’s Honest Post Hoc test (*p* < 0.05). Bars that do not share a letter within a day are significantly different at a 95% confidence level.

The persistence of *Clostridium* was observed during the digestion period (Figure 4a). There were significant differences (*p* < 0.05) in *Clostridium* numbers across the ISRs on day 0, which impacted the dynamics across ISRs during the digestion period (Figure 4b). The cell numbers ranged from 2.9 log_10_ CFU/mL in ISR 2.00 to 4 log_10_ CFU/mL in ISRs 0.50 and 0.25 on day 0. There was an initial 3 log_10_ decrease in *Clostridium* numbers from 4 log_10_ to 1 log_10_ CFU/mL in ISR 0.25 on day 1, but the numbers increased to 2.5 log_10_ on day 3 and decreased to 2 log_10_ by the end of the digestion. There was also a 1.5 log_10_ decrease in counts in ISR 0.50 from 4.0 log_10_ on day 0 to 2.5 log_10_ CFU/mL on day 27. On the other hand, there was a 2 log_10_ increase in *Clostridium* numbers from 3 log_10_ to 5 log_10_ CFU/mL in ISR 1.00 on day 1, and the cell numbers remained constant until the end of the incubation period. The control and ISR 4 had similar clostridial numbers of 3 log_10_ CFU/mL on days 0 and 1, with about 1 log_10_ increase to 4 log_10_ CFU/mL at the end of the digestion.

#### 3.2.3. *Enterococcus* spp.

**Figure 5:**
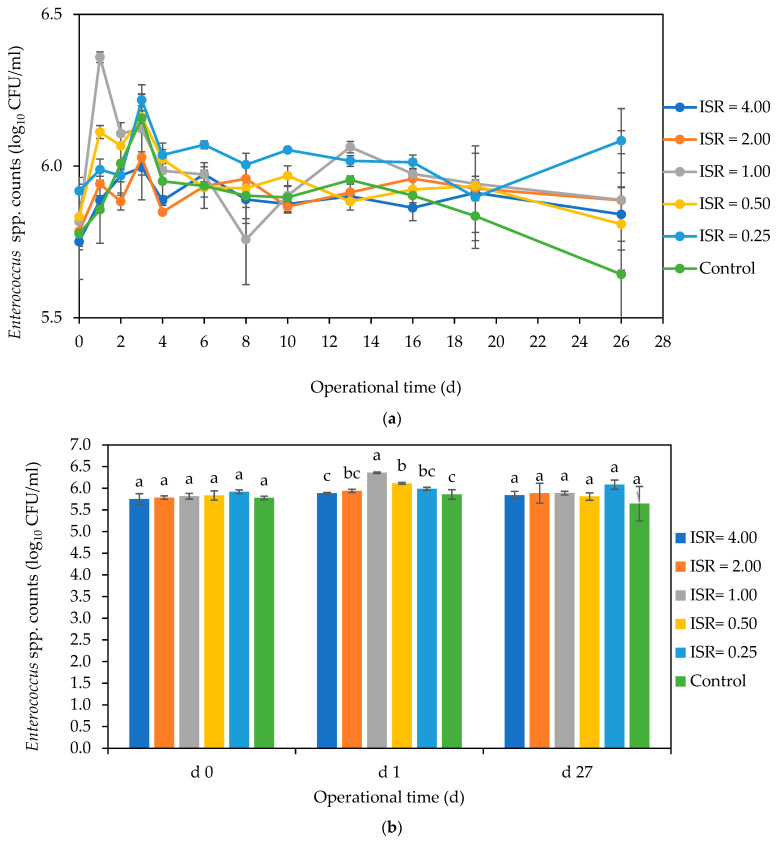
Effect of ISR (4.00, 2.00, 1.00, 0.50, 0.25) and control on *Enterococcus* numbers (log_10_ CFU/mL) during the 27 d anaerobic digestion period (**a**) and on days 0, 1 and 27 (**b**). The data points and bars represent the mean and standard deviation of 4 determinations. The letters on the bars are results obtained from Tukey’s Honest Post Hoc test (*p* < 0.05). Bars that do not share a letter within a day are significantly different at a 95% confidence level.

*Enterococcus* spp. persisted in all the ISRs throughout the digestion period, with no definite reduction pattern (Figure 5a). There were no significant differences (*p* ≤ 0.05) in the *Enterococcus* numbers across the ISRs on days 0 and 27 (Figure 5b). But there was a 0.5 log_10_ increase in its numbers in ISR 1.00 and about a 0.3 log_10_ increase in ISRs 2.00 and 0.50 on day 1.

### 3.3. Total Culturable Aerobic Bacteria

**Figure 6:**
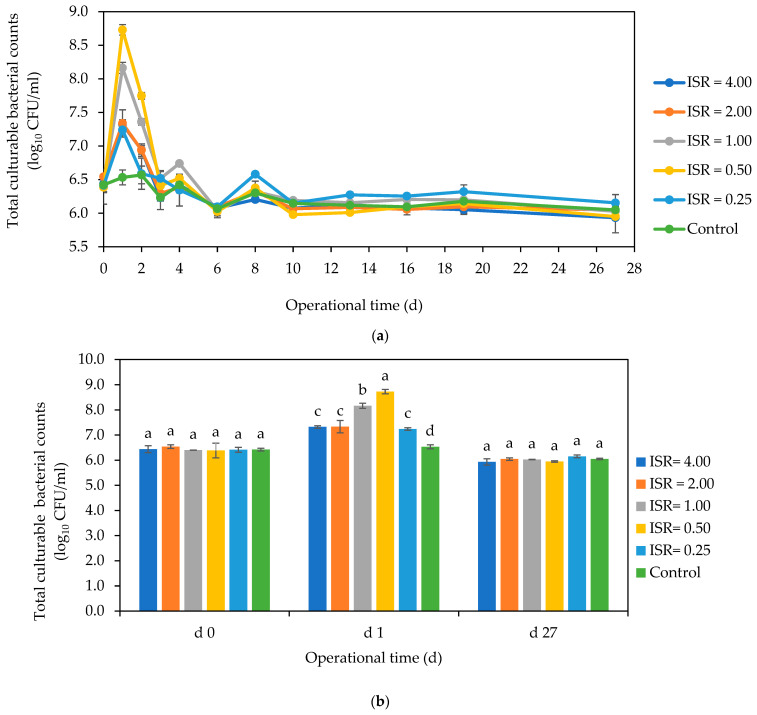
Effect of ISR (4.00, 2.00, 1.00, 0.50, 0.25) and control on total culturable bacteria counts (log_10_ CFU/mL) during the 27 d anaerobic digestion period (**a**), and on days 0, 1, and 27 (**b**). The data points and bars represent the mean and standard deviation of 4 determinations. The letters on the bars are results obtained from Tukey’s Honest Post Hoc test (*p* < 0.05). Bars that do not share a letter within a day are significantly different at a 95% confidence level.

There was an initial increase in total culturable cell numbers across all ISRs on the first day, but the numbers began to decrease on the second day and continued fluctuating till the tenth day (Figure 6a). The culturable bacterial counts reached equilibrium on the tenth day and remained steady at a count of 6 log_10_ CFU/mL for the rest of the digestion period. On day 1, ISRs 0.50 and 1.00 supported more bacterial growth, with a 2.5 log_10_ and 2 log_10_ increase in numbers, respectively, compared to the 1 log_10_ CFU/mL increase in ISRs 4.00, 2.00, and 0.25 (Figure 6b). The control had no significant change in counts throughout the experimental period. At the end of the 27 d digestion period, the total culturable bacterial cell numbers were about 6 log_10_ CFU/mL, with no significant difference across the ISR.

### 3.4. pH Dynamics during Batch AD

**Figure 7:**
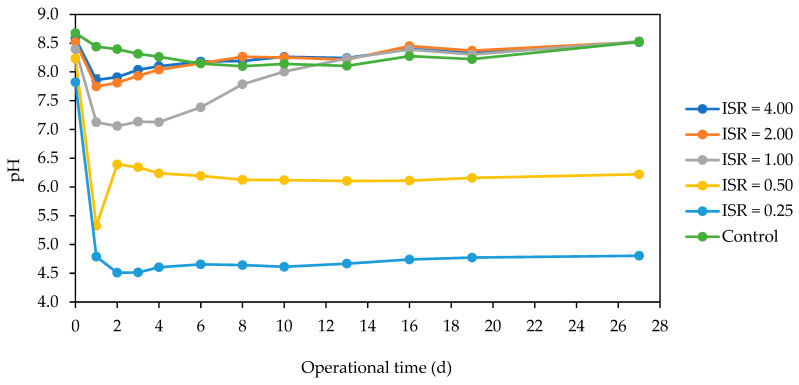
pH dynamics across the ISRs (4.00, 2.00, 1.00, 0.50, 0.25) and control during the 27 d anaerobic digestion period. The data points represent the mean and standard deviation of 4 determinations. The pH data were extracted from Gandhi et al. [25].

There was an initial drop in pH across the ISRs, with an increased magnitude with a decrease in ISR (Figure 7). ISRs 4.00 and 2.00 and the control were in the alkaline range (>7.5) throughout the incubation period. ISR 1.00 had an initial pH close to neutral (7.1) within the 4 days of digestion and increased gradually to above 8.0 by day 10, while ISRs 0.50 and 0.25 had an acidic pH of about 6.5 and 4.5, respectively.

### 3.5. Fate of Clostridium and Enterococcus *spp.* during Secondary Digestion

**Figure 8:**
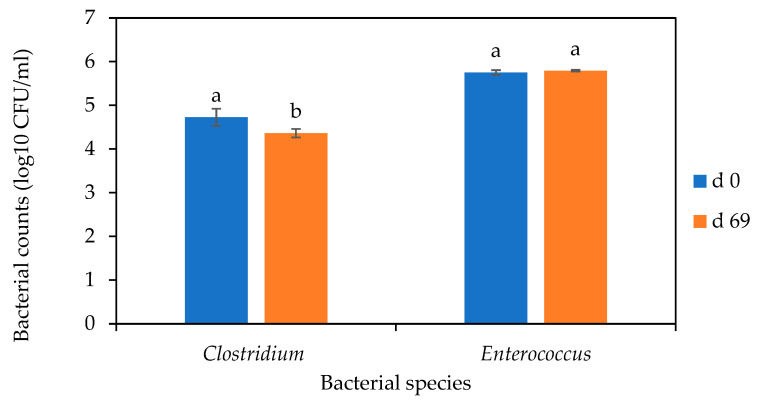
Fate of *Clostridium* and *Enterococcus* after secondary digestion for 69 d. The bars represent the mean and standard deviation of 4 determinations. The letters on the bars are results obtained from Tukey’s Honest Post Hoc test (*p* < 0.05). Bars that do not share a letter within species are significantly different at a 95% confidence level.

After 69 d of digestate storage, there was about 0.4 log_10_ CFU/mL reduction in *Clostridium* numbers (*p* < 0.05), but there was no significant reduction in *Enterococcus* numbers as shown in Figure 8.

### 3.6. Molecular Analyses of Microbial Communities

#### 3.6.1. Identification of Bacterial Strains through Sanger Sequencing

Uniquely re-occurring culturable bacterial strains isolated across all ISRs, and identified through Sanger sequencing are shown in Table 2. Strains of *Bacillus*, *Enterococcus*, and *Klebsiella*, as well as *Escherichia coli* and *Proteus mirabilis* were identified.

#### 3.6.2. Enumeration of Microbial Communities through RT-*q*PCR

##### Bacterial Gene Copies

**Figure 9:**
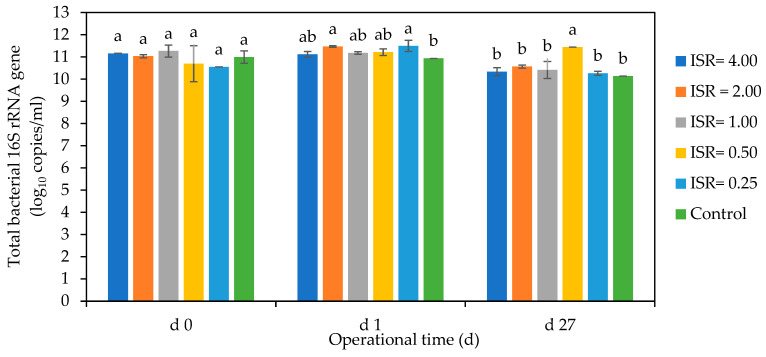
Effect of ISR (4.00, 2.00, 1.00, 0.50, 0.25) and control on bacteria 16S rRNA gene (log_10_ copies/mL) on days 0, 1 and 27 during the 27 d anaerobic digestion period. The bars represent the mean and standard deviation of 4 determinations. The letters on the bars are results obtained from Tukey’s Honest Post Hoc test (*p* < 0.05). Bars that do not share a letter are significantly different at a 95% confidence level.

The bacterial gene copies ranged from 10.5 log_10_ copies/mL in ISR 0.25 to 11.5 log_10_ copies/mL in ISR 1.00 on day 0 and from 10 log_10_ copies/mL in ISR 0.25 to 11.5log_10_ copies/mL in ISR 0.50 on day 27 (Figure 9). On day 1, there was an increase in the bacterial DNA copy number across the ISRs, except ISR 1.00 and 4.00, which experienced a decrease and no change, respectively. ISR 2.00 and 0.25 had significantly higher DNA copies than the others on day 1. The bacterial gene copies decreased across all the ISRs on day 27, except in ISR 0.50, where there was a 1 log_10_ increase in DNA copies/mL. The control experienced a steady decrease in bacterial DNA numbers from day 0 to 27.

##### Fungal Gene Copies

**Figure 10:**
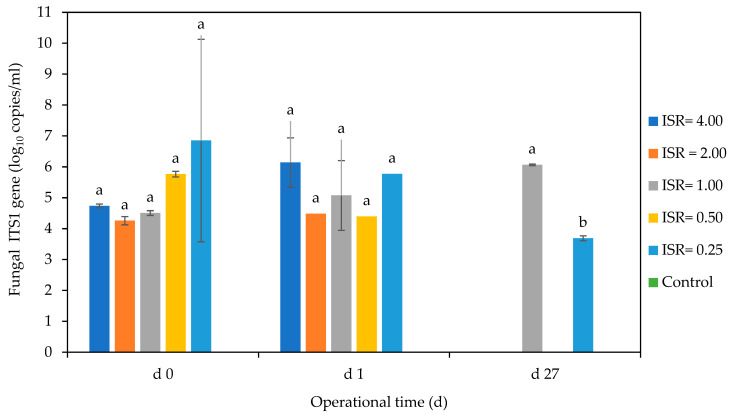
Effect of ISR (4.00, 2.00, 1.00, 0.50, 0.25) and control on fungal ITS1 gene (log_10_ copies/mL) on days 0, 1, and 27 during 27 d anaerobic digestion period. Bars represent the mean and standard deviation of 4 determinations. The letters on the bars are results obtained from Tukey’s Honest Post Hoc test (*p* < 0.05). Bars that do not share a letter are significantly different at a 95% confidence level.

The fungal gene copies were below 6 log_10_ DNA copies/mL in all the ISRs except ISR 0.25, which had the highest DNA of 6.8 log_10_ copies/mL on day 0. Although there were no significant differences (*p* < 0.05) in fungal DNA copies/mL across the ISRs on days 0 and 1, there were increases in the DNA copies in ISR 4.00, 2.00, and 1.00, while there were decreases in the DNA copies in ISR 0.50 and 0.25 on day 1 (Figure 10). On day 27, only ISR 1.00 and 0.25 had detectable fungal DNA copies, and the other ISRs had none. The fungal DNA copies increased steadily across the days in ISR 1.00, while ISR 0.25 experienced a steady decrease. The control had no detectable fungal DNA on all the analysed days.

##### Methanogenic Gene Copies

**Figure 11:**
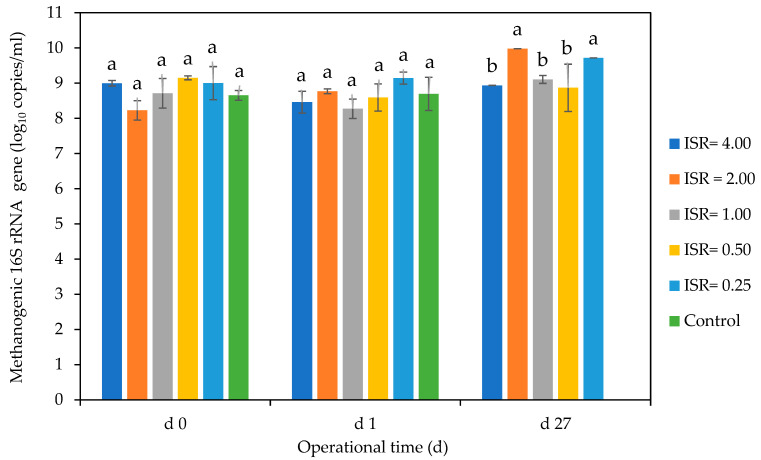
Effect of ISR (4.00, 2.00, 1.00, 0.50, 0.25) and control on methanogenic 16S rRNA gene (log_10_ copies/mL) on days 0, 1, and 27 during 27 d anaerobic digestion period. Bars represent the mean and standard deviation of 4 determinations. The letters on the bars are results obtained from Tukey’s Honest Post Hoc test (*p* < 0.05). Bars that do not share a letter are significantly different at a 95% confidence level.

There were no significant differences in the methanogenic DNA copies across all ISRs on days 0 and 1. The methanogenic DNA increased steadily from 8 log_10_ copies/mL on day 0 to 10 log_10_ copies/mL on day 27 in ISR 2.00 and from 9 log_10_ copies/mL on day 0 to 9.5 log_10_ copies/mL on day 27 in ISR 0.25 (Figure 11). On day 27, ISRs 2.00 and 0.25 had the highest methanogenic DNA of about 10 log_10_. There was no detectable methanogenic DNA in the control on day 27.

## 4. Discussion

### 4.1. Inactivation of E. coli Strains and Coliforms across ISRs during AD of SFW

The RFP-labelled *E. coli* strain which was spiked into the digesters was very susceptible to the AD environment, as it was completely undetectable (4 log_10_ CFU/mL reduction in counts) across all the ISRs and in the control within 3 d. Erickson et al. [31] also reported inactivation of fluorescent-labelled bacterial pathogens by the third day during manure composting. This could be attributed to the low-resistance nature of spiked strains compared with the indigenous ones.

The resident *E. coli* strain was not detected in the substrate or inoculum, but it was detected in ISR 2.00 on d 0, and across the ISRs except ISR 0.25 on d 1. This could be because the strain entered a viable but non-culturable state in the sourced inoculum and regained viability again when nutrients became available. Coupled with other conditions being favourable in the digesters with ISR 4.00 to 0.50, but not in ISR 0.25 due to an overload of substrate, this led to acidification (Figure 7), hence the inability of the strain to thrive at this ISR [25,32]. ISR 0.25 was also the most effective in eliminating coliform bacteria (Figure S1c), with an inactivation rate of 3.5 log_10_ CFU/mL per day, and achieving undetectable levels within 2 d. However, the estimate of the inactivation rate must be regarded as approximate, as it was calculated from the measured concentration of 4.5 log_10_ CFU/mL on day 0 and the estimated limit of detection of 10 CFU/mL below which the d 2 count relied.

The ability of the resident *E. coli* strain to regain viability when the optimum concentration of food was present was corroborated by the lack of detection of the strain at all tested times in the control which was solely inoculum. Zhang et al. [2] also reported that enteroaggregative *E. coli* was reactivated from the viable but non-culturable state when cultured under favourable conditions of nutrient and temperature. In this study, the resident *E. coli* strain which regained viability at ISR 1.00 grew up to 5 log_10_ CFU/mL, and remained detectable till d 13, and the coliform bacteria also survived for 13 d in this digester. This could be due to conditions being more favourable for growth during the initial d at ISR 1 compared to other ISRs (Figure S1c). The pH within the first 4 d of incubation in ISR 1 was close to neutral (about 7.1) [25], which is the optimum pH of most pathogens. Meanwhile, the lower ISRs (0.5 and 0.25), as well as the higher ISRs (4 and 2), were acidic (<6.0) and alkaline (>8.0) pH, respectively, and hence were more effective in sanitising the SFW. It was observed that the inactivation of *E. coli* and coliforms during the batch AD was more rapid in the acidic digesters, while the digesters with the initial neutral pH (ISR 1) were the slowest to achieve pathogen inactivation. Thus, the accumulation of VFAs which is attributable to high total solids, could have contributed to the inability of the resident *E. coli* to regain viability, as well as rapid coliform elimination [25,33]. This observation also agrees with the work of Arias et al. [26] who reported more pathogen killing with higher total solids during batch AD. Although Fagbohungbe et al. [27] reported an increase in *E. coli* and coliform inactivation only with an increase in ISR during batch AD of human faecal material, in this study, it was observed that ISR < 0.5 or ≥2, which led to high acidity or alkalinity, respectively, during the initial days of digestion would enhance *E. coli* and coliform inactivation during mesophilic AD. This finding would be useful in the development of plug-flow continuous mesophilic AD in the field, whereby the effluent from the acidogenic phase which sanitises the waste is fed into the methanogenic phase to produce biogas.

### 4.2. Fate of Clostridium and Enterococcus *spp.* during AD and Post AD Effluent Storage

Although *Clostridium* species are acclimatised to biogas environments [10,34,35,36] and are known to be involved in the hydrolytic and acidogenic stages of the AD process [4], ISRs 0.25 and 0.50 achieved 2 log_10_ and 1.5 log_10_ decrease in counts, respectively. This also agrees with the work of Fontana et al. [21], who observed a slight decrease in the number of culturable *Clostridium* spores in the digestate compared with the inoculum during continuous mesophilic AD. Also, the growth of *Clostridium* in ISR 1.00 observed in this study could be due to their competitive advantage over other microbial species under favourable conditions such as neutral pH (Figure S1d).

There were no significant decreases in the *Enterococcus* numbers throughout the 27 d incubation period. *Enterococci* are very resistant to environmental stress and treatment [11,37]. The findings from this study agrees with the work of Arias et al. [26], who observed no clear trend in *Enterococcus* inactivation after 75 d of batch mesophilic AD, as well as Do et al. [38], who also reported the resistance of *Enterococcus* during a 90 d batch AD of pig slurry.

In addition, post-AD storage did not achieve inactivation of the two genera. Post-AD sanitary practises of digestate, collectively referred to as processes to further reduce pathogens (PFRPs), which include storage, drying, and composting, have been reported to enhance the production of safe digestate [39,40,41]. But in this study, there were no significant decreases in the clostridial and enterococcal numbers after the 69 d post-D storage. *Clostridium* spp. form spores which are resistant to environmental treatment and disinfection and are hardly inactivated by marginally sub-lethal conditions [23,33,42]. Although *Enterococcus* species do not produce spores, their relative resistance could be due to the presence of a thick layer of peptidoglycan and teichoic acids that selectively prevent toxic substances from entering the cell. Furthermore, their ability to produce biofilms could enhance their resistance to disinfection [43]. These results further demonstrate that residence time alone would not play a significant role in eliminating resistant species in AD effluent.

### 4.3. Bacterial Species Identified through Sanger Sequencing during the AD of SFW

Sanger sequencing revealed that the culturable aerobic population which was frequently isolated on the NA plates during the AD of SFW comprised species of *Bacillus*, *Klebsiella*, and *Enterococcus*. These genera have previously been reported in AD effluent [44,45,46,47]. *Bacillus* species form spores which are resistant to environmental stress [48]. They are also known to produce an array of hydrolytic enzymes which are useful in degrading organic materials [49,50]. Although *Klebsiella* and *Enterococcus* do not form spores, their survival in AD could be linked to their ability to form resistant clusters of cells known as biofilms [51,52]. Furthermore, *Enterococcus* is known for its adaptability to stressful environments and complex ecologies due to its ability to survive a wide range of temperatures, pH levels, and oxygen and nutrient concentrations [53,54,55]. *Klebsiella* is a genus of fermentative species that has been linked with lignin degradation [56] and thus is expected to be found in the AD of food waste. Hence, the occurrence of these species in AD could be linked to their tolerance to a diverse range of conditions found in AD and their functional roles in lignocellulose bioconversion.

### 4.4. The Effect of ISR on Total Bacterial and Fungal Communities

The different ISRs showed varying effects on total bacterial and fungal numbers. Food waste is a rich source of nutrients such as carbon and nitrogen for microbial species; thus, the initial increase observed in the total culturable bacterial numbers across the ISRs on day 1 could be due to the growth of the population as they utilised the food substrate [57]. The subsequent stationary culturable bacterial numbers observed from day 10 onwards could be due to the diminishing levels of nutrients, as well as microbial competition, as different species grow and multiply [58]. The importance of the substrate to bacterial growth is further demonstrated in the control, which was the inoculum without substrate and had no increase in total culturable numbers or DNA copies throughout the incubation period. This could be attributed to insufficient nutrient sources for the organisms to grow on [59]. Although ISRs 0.50 and 1.00 showed a higher increase in 2 log_10_ in culturable bacterial numbers on day 1, the bacterial DNA copies were higher in ISRs 2.00 and 0.25. This disparity in the culturable counts and DNA copies observed in the ISRs could be because the digesters selectively enriched different groups of organisms (culturable and non-culturable). Since *q*PCR takes account of the bacterial DNA of aerobic and anaerobic species, whether viable or non-viable, whereas the plate count focused on only culturable aerobic bacteria [60], this could be responsible for the observed pattern in culturable bacterial counts and DNA copies.

Although fungi are generally aerobic organisms which are not expected to grow under anaerobic conditions, some anaerobic fungal species in the phylum Neocallimastigomycota have been reported to grow in AD environments where they have improved biogas production [61,62]. The ability of fungi to survive in AD could also be linked to their ability to form resistant spores [63]. Fungi produce an array of hydrolytic enzymes which aid in the degradation of complex lignocellulosic materials, which are abundant in food waste [64]. In this study, ISR 0.25 had the highest fungi DNA copies on day 0. This could be due to the high substrate concentration at this ISR, as fungi are known to growth optimally under high nutrient concentration, and reduced water activity [65,66]. At the end of the digestion period, the bioreactors with high substrate concentrations—ISR 0.50 and 0.25—achieved about 6 log_10_ and 3 log_10_ deceases in fungal ITS1 gene copies/mL, respectively. This reduction could be due to the effect of VFAs on fungi, as also observed with bacteria in this study. It could also be due to the limited survival of facultative anaerobic fungi under anaerobic conditions [63,67]. The survival in ISR 1 could be due to favourable pH and nutrient conditions in these bioreactors. We observed in this study that batch mesophilic AD was able to achieve up to a 6 log_10_ reduction in fungal DNA copies in the digesters with the initial acidic and alkaline conditions. This could be an indication that fungal persistence may not be an issue in AD effluent when operating conditions are properly optimised.

### 4.5. Effect of ISRs on Methanogenic Gene Copies

Enumerating the population of methanogens during AD could be used as an indicator of biogas production [68]. Methanogens are very sensitive to environmental conditions, and thus, optimum nutrient concentration and favourable pH would support their growth. Studies revealed that high ISR favoured methanogenesis, and this could be an indication of high methanogenic population [27,69].

In this study, *q*PCR detection of Methanogenic 16S rRNA gene showed that ISR 2.00 had a steady increase in methanogenic population from d 0 to 27, which could be a putative indication of methane production. This agrees with the finding of Gandhi et al. [25], who reported the highest methane production in ISR 2.00. The lack of detectable methanogenic 16S rRNA in the control on d 27 could be due to lack of usable nutrient for the growth of the methanogenic population [70], coupled with degradation of the initially present methanogenic DNA [71].

## 5. Conclusions

This study has demonstrated that ISR influences the pH of the digesters, which in turn determines pathogen inactivation and microbial kinetics during the anaerobic digestion of food waste. Treating food waste using batch mesophilic AD under optimised conditions can achieve its sanitisation before recycling as fertiliser of disposal in the environment. Rapid pathogen inactivation can be linked to acidic pH due to the accumulation of toxic organic acids at high substrate concentrations. This finding would be useful in optimising the acidogenic phase of two-phased acidogenic–methanogenic mesophilic AD. The pH during the initial days of incubation played a role in the growth or inactivation of pathogens. The lower ISRs (0.50 and 0.25) and higher ISRs (4.00 and 2.00) gave rise to acidic and alkaline pH, respectively, while ISR 1 had neutral pH. Pathogen removal was achieved more rapidly in the acidic and alkaline digesters, while the neutral digesters were not only the slowest at pathogen inactivation, but they also supported clostridial and fungal growth due to pH being close to neutral within the initial period of the digestion. This study showed that running AD at ISRs which could lead to neutral pH (ISR 1.00) can result in the growth and proliferation of resistant putative human pathogens. *Enterococcus* species persisted throughout the digestion period, irrespective of the ISRs. This means that the inactivation potency of batch AD varies with the pathogen species. *q*PCR enumerated the DNA copies of total bacterial and fungal, as well as methanogens, which are generally difficult to isolate using culture-based methods. This makes it possible to putatively link microbial species to functional roles during AD. Fungi do not thrive well in batch AD and can be removed beyond detection levels in acidic and alkaline bioreactors. Future studies would benefit from exploring the metagenomic approach to quantitatively monitor the dynamics in pathogen and microbial community structure during batch mesophilic AD of food waste.

## Figures and Tables

**Table 1:** Background bacterial counts (log_10_ CFU/mL) of substrate (SFW) and inoculum used for batch AD.

Bacteria	SFW (log_10_ CFU/mL)	Inoculum (log_10_ CFU/mL)
Total culturable bacteria	5.57 ± 0.12	6.34 ± 0.02
*E. coli*	-	-
Total coliforms	4.47 ± 0.05	3.26 ± 0.24
Non-lactose fermenting species (on MacConkey agar)	-	5.75 ± 0.05
*Clostridium* sp.	-	3.37 ± 0.01
*Enterococcus* sp.	2.06 ± 0.08	6.90 ± 0.08

**Table 2:** Bacterial strains isolated from all the bioreactors and identified using Sanger sequencing.

Bacterial Strains	Accession Number
*Escherichia coli* DSM 30083 = JCM 1649	CP033092.2
*Klebsiella pneumoniae* A17	KU711920.1
*Enterococcus dispar* GS02	KY569462.1
*Bacillus aerius* SPF29	MH160719.1
*Klebsiella michiganensis* F107	CP024643.1
*Bacillus* sp. M-265	MK634697.1
*Enterococcus faecium* NMCC-203	MN493726.1
*Bacillus encimensis* RW13	KY569474.1
*Proteus mirabilis* LO01	KX966458.1

## Data Availability

Data is contained within the article and Supplementary Materials.

## Supplementary Materials

The following supporting information can be downloaded at https://www.mdpi.com/article/10.3390/microorganisms12030603/s1. Table S1: Recipe for simulated food waste used in the batch AD process based on household food waste in the UK. Table S2: Substrate (simulated food waste) and inoculum characteristics. Table S3: Primers used for *q*PCR. Figure S1: Effect of ISR on pH and bacterial counts showing how ISR influenced pH, which in turn determined the inactivation of RFP-labelled *E. coli*, resident *E. coli*, coliforms, and *Clostridium* during the first 3 days of AD.
