# Supplementary material for: Effect of the Inoculum-to-Substrate Ratio on Putative Pathogens and Microbial Kinetics during the Batch Anaerobic Digestion of Simulated Food Waste
Source: Microorganisms. 2024 Mar 18;12(3):603. doi: 10.3390/microorganisms12030603 (PMC10974213; doi:10.3390/microorganisms12030603)
Supplement: Supplementary file 1 [file microorganisms-12-00603-s001.zip › microorganisms-2807731-supplementary.pdf]

Supplementary material

Effect of inoculum to substrate ratio on putative pathogens and microbial kinetics during batch anaerobic digestion of simulated food waste

Saanu Victoria Otite [1], Bhushan P. Gandhi [2], Esther Agyabeng Fofie [1], Alfonso José Lag-Brotons [2], Lawrence I. Ezemonye [3,4], Alastair D. Martin [2], Roger W. Pickup [5] and Kirk T. Semple [1,*]

[1] Lancaster Environment Centre, Library Avenue, Lancaster University, Lancaster, LA1 4YQ, UK
[2] Engineering Department, Gillow Avenue, Lancaster University, Lancaster, LA1 4YW, UK
[3] Centre for Global Eco-innovation Nigeria, University of Benin, Benin City, Edo State, PMB 300313, Nigeria
[4] Igbinedion University Okada, Benin City, Edo State, PMB 0006, Nigeria
[5] Division of Biomedical and Life Sciences, Furness Building, Lancaster University, Lancaster, LA1 4YG, UK

***** Correspondence: k.semple@lancaster.ac.uk; Tel.: +44 (0)1524 510554

Table S1: Recipe for simulated food waste used in the batch AD process based on household food waste in the UK (WRAP, UK (Ventour, 2008)). Culled from Gandhi *et al*. (2022)

**Household food waste in UK (WRAP, 2007)**		**Simulated household food waste**	
**Food group**	**% Weight (fresh basis)**	**Food group**	**% Weight (fresh basis)**
Bakery	13.4%	Sainsbury's Plain Naan	13.80%
Vegetables	25.8%	Bird Eye Mixed Vegetables	26.57%
Mixed foods	10.5%	Sainsbury's Fish Pie	10.81%
Fruit	16.4%	Sainsbury's Fruit Platter	16.89%
Meat and fish	8.4%	Sainsbury Beef Mince 12% Fat	8.65%
Salad	4.4%	Sainsbury's Bisto Salad	4.53%
Dairy	3.5%	Sainsbury's Woodland Free Range Large Eggs	3.61%
Dried food	2.5%	Sainsbury's Penne	2.58%
Drinks	8.0%	Summerfruits Juice Drinks	8.24%
Condiments	2.4%	Heinz Tomato Ketchup	2.4%
Confectionery	1.0%	Cadbury Dairy Milk Chocolate	1.0%
Desserts	0.8%	Bramley Apple Pies	0.8%
Other	3.0%	----------------	------------
Total	100%	Total	100%

Table S2: Substrate (simulated food waste) and inoculum characteristics (mean ± standard deviation) Culled from Gandhi *et al*. (2022)

Parameter	Substrate	Inoculum
pH	5.80 ± 0.00	8.64 ± 0.01
TS (%)	19.75 ± 0.09	6.58 ± 0.08
VS (% dry basis)	94.97 ± 0.11	60.87 ± 0.29
VS (% wet basis)	18.75 ± 0.09	4.01 ± 0.05
Carbohydrates (% wet basis)	11.16 ± 0.16	0.48 ± 0.05
Proteins (% wet basis)	3.21 ± 0.06	1. 61 ± 0.04
Lipids (% wet basis)	2.64 ± 0.01	0.69 ± 0.04
Total VFAs (g/l)	4.49 ± 0.14	0.74 ± 0.06
Total ammonia nitrogen (mg/l)	124 ± 12	4223 ± 19
Partial alkalinity (as, g $CaCO_3$/l)	0.36 ± 0.07	17.38 ± 0.05
Intermediate alkalinity (as, g $CaCO_3$/l)	2.08 ± 0.06	4.82 ± 0.06
Total alkalinity (as, g $CaCO_3$/l)	3.08 ± 0.14	22.76 ± 0.04
Cellulose (% dry basis)	4.01 ± 0.18	9.27 ± 0.43
Lignin (% dry basis)	7.16 ± 0.44	12.20 ± 0.02
C (% dry basis)	47.32 ± 0.23	34.27 ± 0.09
H (% dry basis)	6.75 ± 0.04	4.71 ± 0.02
N (% dry basis)	3.42 ± 0.02	4.37 ± 0.02
S (% dry basis)	0.37 ± 0.08	0.82 ± 0.02

Table S3. Primers used for *q*PCR

Target group	Primer name	Target region	Sequence	Amplicon size
Bacteria	341 F	16S rRNA	CCTACGGGAGGCAGCAG	434
	806 R		GGACTACHVGGGTWTCTAAT	
Fungi	Lwin F	ITS1	GAGGAAGTAAAAGTCGTAACAAGGTTTC	120
	Lwin R		CAAATTCACAAAGGGTAGGATGATT	
Methanogens	Met1 F	16S rRNA	GGATTAGATACCCSGGTAGT	191
	Met1 R		GTTGARTCCAATTAAACCGCA	

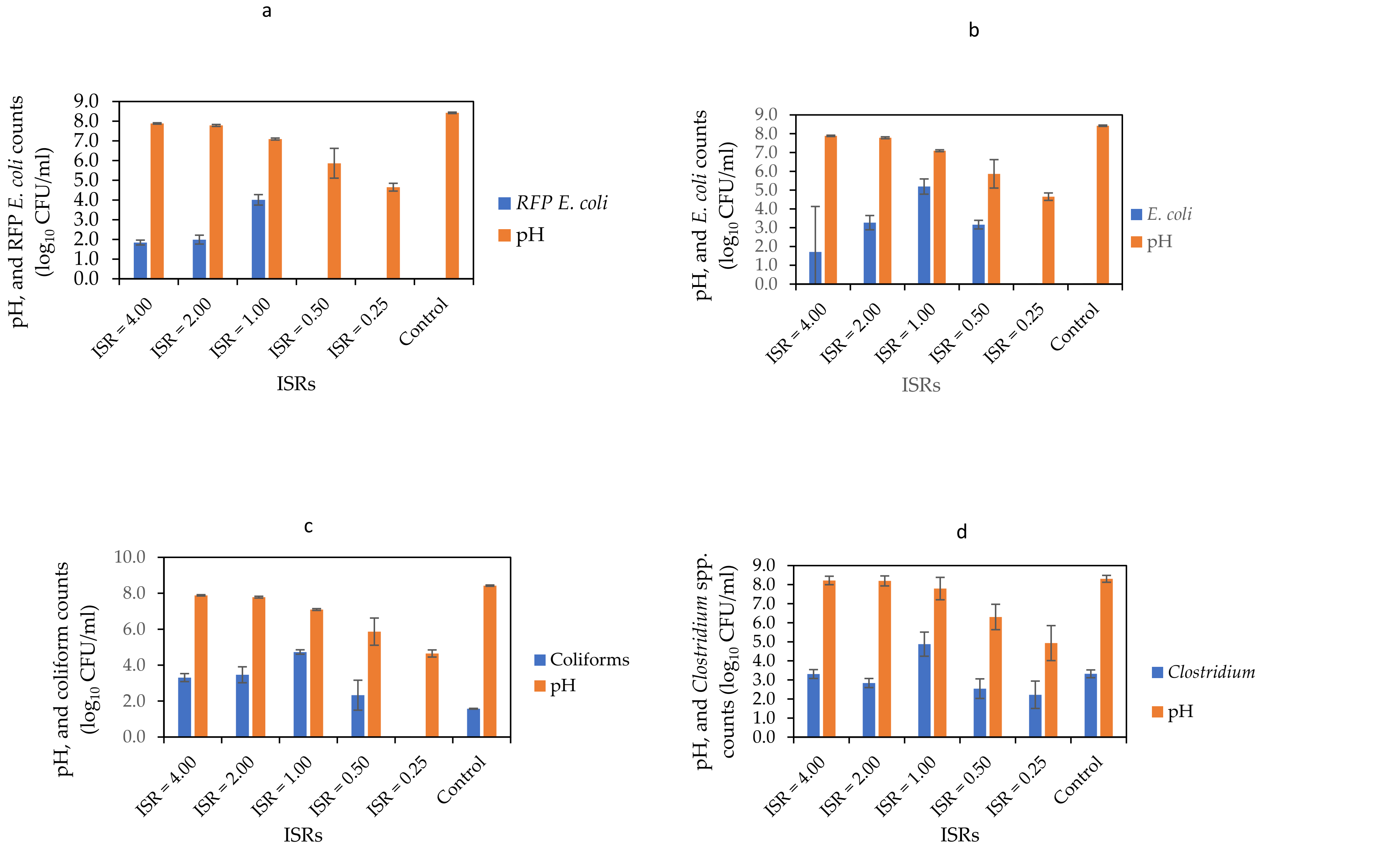

Figure S1: Effect of ISR on pH and bacterial counts showing how ISR influenced pH which in turn determined the inactivation of RFP-labelled *E. coli* (a) resident *E. coli* (b) coliforms (c) and *Clostridium* (d) during the first 3 days of AD. Data points are mean and standard deviation of pH and bacterial counts obtained during the first 3 days of AD.
